# Targeting the Microenvironment in MDS: The Final Frontier

**DOI:** 10.3389/fphar.2020.01044

**Published:** 2020-07-09

**Authors:** Patric Teodorescu, Sergiu Pasca, Delia Dima, Ciprian Tomuleasa, Gabriel Ghiaur

**Affiliations:** ^1^ Department of Hematology, Iuliu Hategan University of Medicine and Pharmacy, Cluj-Napoca, Romania; ^2^ Department of Oncology, The Johns Hopkins Hospital, Johns Hopkins Medicine, Baltimore, MD, United States

**Keywords:** myelodyslastic syndromes, microenvironment, azacytidine, lenalidomide, luspatercept, rigosertib, all-trans retinoic acid, CYP26 enzymes

## Abstract

Myelodysplastic syndromes (MDS) are a heterogeneous group of malignant disorders of hematopoietic stem and progenitor cells (HSPC), mainly characterized by ineffective hematopoiesis leading to peripheral cytopenias and progressive bone marrow failure. While clonal dominance is nearly universal at diagnosis, most genetic mutations identified in patients with MDS do not provide a conspicuous advantage to the malignant cells. In this context, malignant cells alter their adjacent bone marrow microenvironment (BME) and rely on cell extrinsic factors to maintain clonal dominance. The profoundly disturbed BME favors the myelodysplastic cells and, most importantly is detrimental to normal hematopoietic cells. Thus, the MDS microenvironment not only contributes to the observed cytopenias seen in these patients but could also negatively impact the engraftment of normal, allogeneic HSPCs in patients with MDS undergoing bone marrow transplant. Therefore, successful therapies in MDS should not only target the malignant cells but also reprogram their bone marrow microenvironment. Here, we will provide a synopsis of how drugs currently used or on the verge of being approved for the treatment of MDS may achieve this goal.

## Introduction

Myelodysplastic syndromes (MDS) are a heterogeneous group of malignant disorders of hematopoietic stem and progenitor cells (HSPCs), mainly characterized by ineffective hematopoiesis leading to peripheral cytopenias and progressive bone-marrow failure. Moreover, these are also associated with a high risk of progression to acute myeloid leukemia (AML). MDS is the most commonly diagnosed myeloid malignancy in the United States (DeZern, 2015). The median age at diagnosis is 72 years old. These patients are fragile and suffer from multiple comorbidities. Therefore, they are often ineligible for bone marrow transplantation, the only curative therapeutic option in MDS. The 3-year overall survival rate of 35–45% (Rollison et al., 2008) highlights the need for novel therapies in patients with MDS.

Numerous genetic events have been implicated in the pathogenesis of MDS. These mutations range from large chromosomal abnormalities such as deletions/additions (i.e. del(5q), del(7q)) to specific gene mutations affecting various biological processes including: spliceosome (i.e. SF3B1, SRSF2); transcription factors (i.e. RUNX1, ETV6); or DNA/chromatin epigenetic changes (i.e. TET2, DNMT3a, ASXL1). While clonal dominance is nearly universal in MDS at diagnosis, these mutations don't provide a conspicuous advantage to the malignant cells. In most cases, the malignant clone continues to coexist alongside normal hematopoietic stem cells, which are somehow inhibited (Calvi et al., 2019). These observations led to the hypothesis that MDS cells get an extrinsic support, from the mesenchymal stromal cells (MSCs) in the bone marrow microenvironment (BME). The interaction between the mutant clone and BME plays an important role in disease homeostasis. Since the main causes of death in MDS are cytopenia-related complications (infections, hemorrhage), restoring the function of residual normal hematopoiesis is a major goal in the treatment of MDS.

## The Bone Marrow Microenvironment in MDS

Research in MDS has been hindered by lack of available models. More so, as MDS cells are difficult to study ex vivo due to very high rate of apoptosis. Support from BME proved to be essential to maintain some MDS cells *ex vivo*. However, the mechanisms through which this support is provided is currently not fully understood. To this end, bone marrow derived MSCs from patients with MDS (MDS-MSCs), and not from normal individuals, are uniquely effective in maintaining the MDS clones (Medyouf et al., 2014). This observation led to the hypothesis that the BME contributes to MDS pathogenesis, homeostasis, and even response to treatment.

Regarding pathogenesis, several studies have shown that MDS and other myeloproliferative neoplasms (MPNs) can be initiated by modifications in the BME. The deletion of various genes such as Dicer1, Sipa1, Retinoblastoma protein (Rb), and Retinoic Acid Receptor gamma (RARy), as well as the activation of the Hedgehog pathway through the knockout of PTCH2, have been reported to lead to the development of MDS or MPNs in mice (Walkley et al., 2007a; Walkley et al., 2007b; Raaijmakers et al., 2010; Klein et al., 2016; Xiao et al., 2018). However, these specific mutations are found only in selected cases of MDS patients and their involvement in human pathogenesis is yet to be clarified.

**Figure 1:**
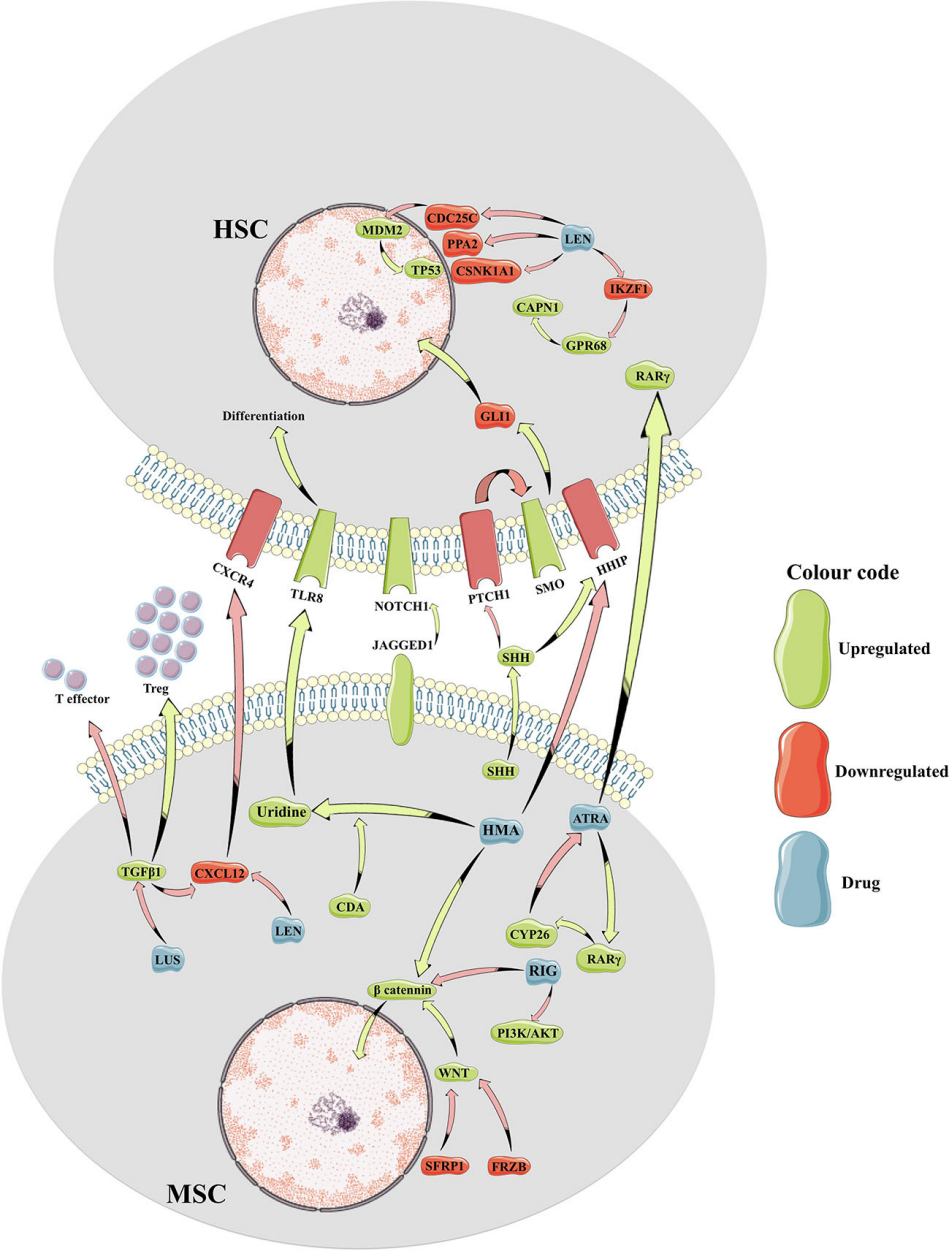
Cartoon representation of molecular crosstalk between mesenchymal bone marrow microenvironment and the myelodysplastic hematopoietic cells. HSC, hematopoietic stem cell; MSC, mesenchymal stem cell; Treg, T regulatory cells; HMA, hypomethylating agents; LEN, lenalidomide; LUS, luspatercept; RIG, rigosertib; ATRA, all-trans retinoic acid; CAPN1, calcium-dependent protease calpain1; CDA, cytidine deaminase; CDC25C, Cell Division Cycle 25C gene; CSNK1A1, casein-kinase 1A1; GPR68, G Protein-Coupled Receptor 68 gene; IKZF1, IKAROS Family Zinc Finger 1 gene; PI3K, Phosphatidylinositol-3 Kinase; PPA2, Inorganic Pyrophosphatase gene; RARγ, Retinoic Acid Receptor Gamma; SHH, Sonic Hedgehog ligand; TGFβ, transforming growth factor beta; TLR8, Toll-Like Receptor 8.

**Figure 2:**
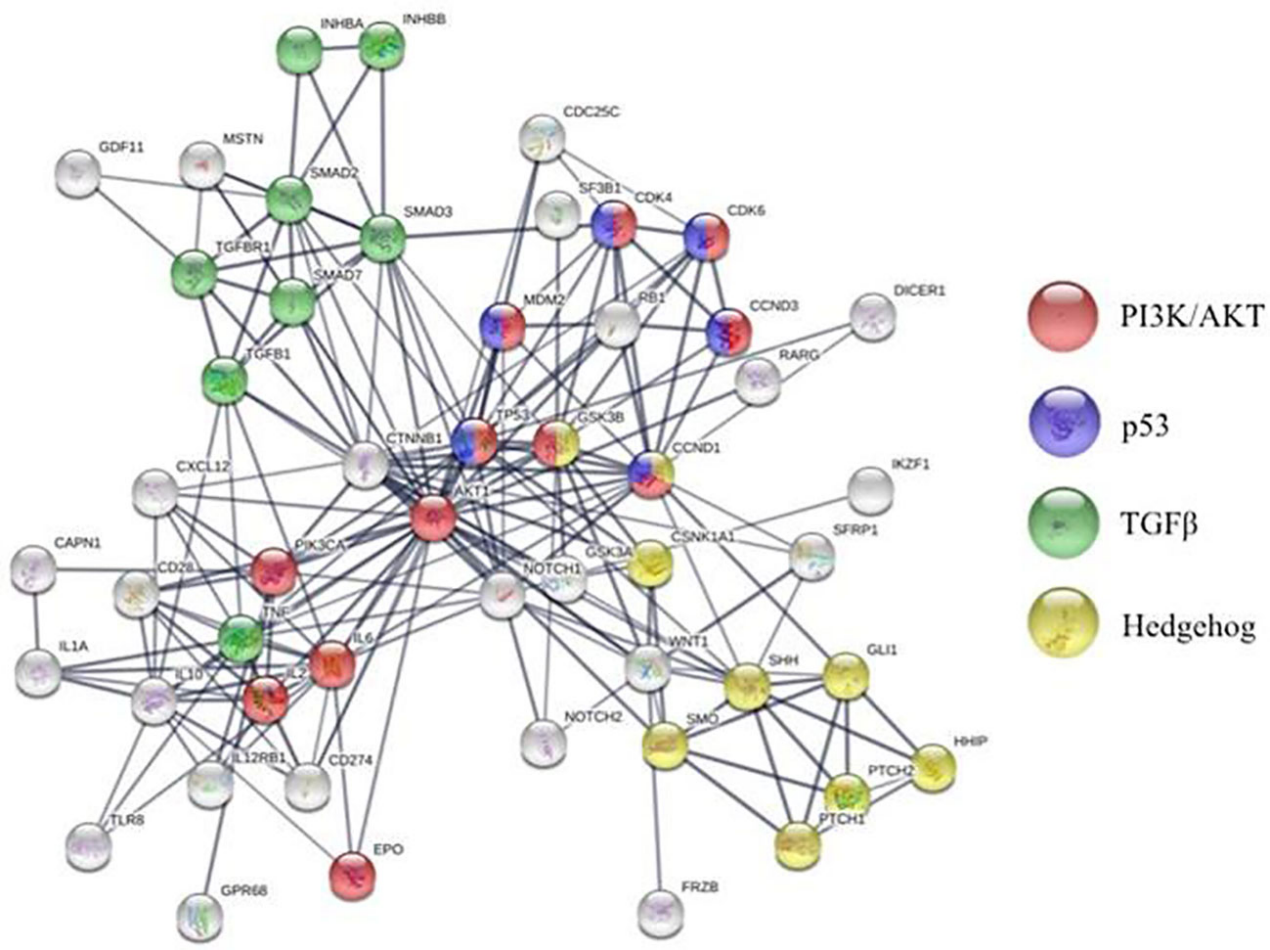
STRING analysis of molecular pathways altered in bone marrow microenvironment of patients with MDS compared to healthy individuals.

Bidirectional crosstalk between the MDS clone and their surrounding milieu not only maintains the malignant clone but also reshapes the BME (**Figure 1**). As a result, MSCs from patients with MDS are reprogramed to promote maintenance of the malignant clone at the expense of normal hematopoiesis (Geyh et al., 2013). To this end, MSCs derived from patients with MDS (MDS-MSCs) display morphological changes *ex vivo* (Ferrer et al., 2013; Falconi et al., 2016), impaired growth capacity, increased senescence, decreased osteogenic differentiation, and overall decreased survival (Geyh et al., 2013). The mechanisms responsible for these alterations are only partly characterized. For instance, over secretion of alarmins, such as S100A9 and S100A8, by the MDS cells activates the inflammasome in the MSCs (Chen et al., 2016) leading to aberrant activation of various molecular programs resulting in higher secretion of cytokines such as interferons and IL32 (**Figure 2**) (Kim et al., 2015; Zhang et al., 2016). Also, the secretion of extracellular vesicles containing miR-7977, by the MDS cells, was shown to reduce the hematopoietic supporting capacity of MSCs. This was achieved through the reduction of several hematopoietic growth factors such as Jagged-1, stem cell factor, and angiopoietin-1 (Horiguchi et al., 2016). In addition, several *in vitro* studies suggest that MDS-MSCs have impaired PI3K/AKT and Wnt/ß-catenin signaling (Pavlaki et al., 2014; Falconi et al., 2016) which may explain their abnormal proliferation, self-renewal, and osteogenic differentiation (**Figure 2**) (Boland et al., 2004; Glass et al., 2005). To this end, high endogenous erythropoietin levels often seen in MDS patients may downregulate Wnt pathway and impair osteogenic differentiation of MDS-MSCs (Balaian et al., 2018). In this context, the wide use of erythropoietin and erythropoiesis-stimulating agents may inadvertently impact the BME in patients with MDS. On the other hand, in murine models of MDS, Wnt/ß-catenin pathway is hyperactive in MSCs (Kode et al., 2014; Bhagat et al., 2017) and is capable of disease initiation through overexpression of Notch-ligand, Jagged1 (Kode et al., 2014). It is currently unknown whether or not activation of Wnt/ß-catenin pathway plays distinct roles in disease initiation *vs.* maintenance or if the observed differences are due to unique features of the models used (mouse *vs.* human). Nevertheless, MDS-MSCs have low levels of Wnt pathway antagonists (FRZB and SFRP1) likely due to their hyper methylation explaining the upregulated Wnt/ß-catenin signaling (**Figure 1**) (Bhagat et al., 2017). While disrupted methylation profiles in the MDS hematopoietic clones are well characterized, MDS-MSCs also display numerous differentially methylated genes explaining their cellular phenotype and transcriptional regulation (**Figure 2**) (Geyh et al., 2013). Among such genes, human Hh-interacting protein gene (HHIP) was shown to be hyper methylated in MDS-MSCs (Kobune et al., 2012). Low expression of HHIP and the associated activation of the Hedgehog pathway in MDS-MSCs are important for the survival of the MDS clone (**Figure 1**). Such complex changes in MDS-MSCs make them more suitable to support the MDS clone perhaps at the expense of normal hematopoiesis. To this end, MDS-MSCs create an inflammatory milieu that is detrimental to healthy HSPCs (Muto et al., 2020). On the other hand, MDS-HSPCs gain competitive advantage in this inflammatory environment by activating their non-canonical NF-kB pathway *via* Traf6. In addition, the SDF-1CXCR4 axis is also dysregulated in MDS. Studies have found correlations between higher levels of SDF-1 in low-grade MDS and increased apoptosis of hematopoietic cells, and higher levels of CXCR4 and increased bone-marrow angiogenesis in high-grade MDS (Zhang et al., 2012).

The mechanisms by which the corrupt MDS-MSCs signal to the MDS clone are diverse and only beginning to be explored. For instance, MDS-MSCs release high quantities of small extracellular vesicles (sEVs) loaded with miR-486-5p (Meunier et al., 2020). This miR-486-5p can not only promote leukemogenesis and is overexpressed in leukemic cells of Down syndrome patients (Shaham et al., 2015) but also may induce oxidative stress and apoptosis of normal HSPC and senescence in MSCs (Kim et al., 2012). Higher secretion of sEVs containing miR10a and miR15a by MDS-MSCs increase the viability and clonogenicity of MDS HSPCs (Muntión et al., 2016).

Lastly, MDS-MSCs secrete high levels of TGFß1 (Zhao et al., 2012), a cytokine profoundly immunosuppressant for B, T, and NK cells and immunostimulating for regulatory T cells (Treg) (Cagliani et al., 2017). Thus, the high levels of TGFß1 seen in patients with high risk MDS (Zhao et al., 2012) promote a immunosuppressive microenvironment with reduced CD4+ T-cell population, CD8 T-cell exhaustion, a significant decrease in NK activating receptors, and an increase of non-cytotoxic NK-cells (CD56bright) (**Figure 1**) (Montes et al., 2019).

In spite of recent advances in our understanding of clonal architecture and mutational landscape in MDS, overall prognosis of these patients remains poor. More so, therapeutic responses are transient at best without the use of bone marrow transplantation. This may be explained by the functional heterogeneity within the MDS clone. For instance, some MDS cells may be protected from therapy by the MDS-MSCs and be responsible for disease relapse (**Figure 1**). To this end, BM MSCs express high levels of cytidine-deaminase (CDA), an enzyme that metabolizes azacitidine and decitabine, two essential drugs in the treatment of MDS (Alonso et al., 2015; Su et al., 2019). Thus, the pharmacokinetics of these drugs may be altered in the BME. On the other hand, molecular changes induced in the MDS clone need to be reinforced by reprogramming the BME in order to be sustained. To this end, abnormal methylation patterns observed in MDS-MSCs for instance may also be amenable to correction by the current therapeutic tools used in these patients.

Here, we will provide a synopsis of how drugs currently used or on the verge of being approved for treatment of MDS may impact not only the mutant hematopoietic cells but also the surrounding microenvironment.

## Current Therapies

### Hypomethylating Agents

Epigenetic therapy with 5-azacitidine (5-Aza) or decitabine represents a stepping stone in the treatment of MDS and remains the only FDA approved option for high-risk disease.

5-Azacitidine and decitabine work as hypomethylating drugs and are related molecules with similar structures and overlapping effects. They both incorporate into the DNA of cells resulting in depletion of intracellular methyltransferases (DNMTs) and reversal of altered methylation patterns of MDS cells. As such, it takes four to six cycles of therapy to reach full clinical responses to azanucleotides in MDS. More often than not the responses correlate with improvement of cytopenias but no significant changes in the clonal architecture suggesting some correction of the differentiation profile for the MDS clone rather than elimination of the malignant cells. To this end, treatment with azanucleotides resembles classical differentiation therapy seen with all-trans retinoic acid (ATRA) in acute promyelocytic leukemia (APL) (Stresemann and Lyko, 2008; Gnyszka et al., 2013). In general, 30–50% of patients show clinical benefit from treatment with azanucleotides but their response is short lived. It is currently unknown why only some patients benefit from treatment and even in those that respond, why the malignant clone is not completely eliminated (Diesch et al., 2016; Ball et al., 2017). A plausible explanation is that these drugs have a blunted effect on the highly quiescent MDS stem cells since epigenetic changes need dividing cells to take effect (Suarez and Gore, 2013). Also, since stroma cells in the bone marrow expresses CDA (Alonso et al., 2015), the enzyme that inactivates 5-Aza, perhaps impaired pharmacokinetics in bone marrow niches may play a role (**Figure 1**). To this end, cell cultures that express high levels of CDA are less sensitive to 5-Aza (Mahfouz et al., 2013). However, a study conducted on a small cohort of patient samples, showed that high levels of CDA after treatment, might be an indicator of responsiveness (Murakami et al., 2019). Nevertheless, CDA is necessary for the transformation of HMAs to uridine, an activator of the toll-like receptor 8 (TLR8) with potential implications for the impaired myeloid differentiation seen in MDS (**Figure 1**) (Ignatz-Hoover et al., 2015; Miyake et al., 2017; Furusho et al., 2019). Some suggest that the degree of myeloid differentiation of the MDS clone prior to treatment with azanucleotides is a better indicator for response to therapy than the previously proposed abnormal methylation of endogenous retro-elements (Kazachenka et al., 2019). While cell intrinsic properties of the MDS clone can certainly dictate the differentiation state of these cells, an altered BME is bound to play an essential role. Since MDS-MSCs have different methylation patterns compared to their healthy counterparts (Geyh et al., 2013), it begs the question if HMA-induced changes in the BME contribute to the clinical responses observed. To this end, 5-Aza enhances the ability to support normal hematopoiesis in MDS-MSCs, while decreasing their support for MDS HSPCs (Wenk et al., 2018; Poon et al., 2019). During disease homeostasis, MDS-induced impairment of MSCs' functions appears reversible by 5-Aza but only up to a certain point in disease progression (Poon et al., 2019). 5-Aza restored the hematopoiesis-supporting capacity of most primary MDS-MSCs samples derived from low grade MDS but not those from advanced-stage disease (Poon et al., 2019). However, treatment with 5-Aza is capable to enhance hematopoiesis-supporting properties of healthy MSCs as well, suggesting that the effect is not specific to MDS-induced changes (Wenk et al., 2018). It is currently unknown what molecular program induced by azanucleotides rescue MSC function. RNA-seq data suggests that treatment with 5-Aza optimizes interferon signaling and extracellular matrix homeostasis, including collagen type IV and VI with major implications for HSC anchorage, self-renewal, and differentiation (Essers et al., 2009; Gattazzo et al., 2014; Wenk et al., 2018). In addition, treatment of MDS-MSCs with 5-Aza reverses aberrant methylation patterns of these cells resulting in rescue of frequently altered signaling pathways such as over activation of betaβ-catenin and the downregulation of HHIP (**Figure 1**) (Kobune et al., 2012; Bhagat et al., 2017).

Finally, treatment with decitabine *ex vivo* can reset the immunosuppressive phenotype of MDS-MSCs, resulting in decreased expression of PDL1 and optimization of the immune milieu in the BM of patients with MDS (Pang et al., 2019). To this end, decitabine decreases T cell differentiation towards Tregs, a population known to be expanded in high risk MDS (Kordasti et al., 2007; Zhao et al., 2012).

Thus, azanucleotides-induced changes of BME may not only negatively impact the MDS clone but also rescue the residual normal hematopoiesis. A nurturing BME could help in engraftment of donor HSCs and promote hematopoietic reconstitution in the settings of allogeneic bone marrow transplant (alloBMT). Treatment with 5-Aza prior to or post alloBMT may prove beneficial for patients with MDS and remains an area of active research.

### Lenalidomide

Lenalidomide is a derivative of thalidomide that, in addition to its well documented efficacy in the treatment of multiple myeloma, has also proven activity in patients with low risk MDS (LR-MDS) and 5q31-5q32 deletion (List et al., 2006). Treatment with lenalidomide of patients with transfusion dependent LR-MDS with 5q- results in improved transfusion requirements and even cytogenetic complete remission (CR) in some cases. The putative mechanisms of action take into account the unique sensitivity of 5q- MDS cells to lenalidomide and explore the role of haploinsufficiency of various genes in this process. As such, three main haploinsufficient mechanisms have been proposed: a) CDC25C and PPA2 resulting in subsequent modulation of MDM2-TP53 pathway and cell cycle arrest (Wei et al., 2009); b) casein- kinase-1a1 (CSNK1A1) with resultant TP53 induction and clonal arrest (Schneider et al., 2014); and c) activation of calcium dependent protease calpain (CAPN1) due to not only overexpression of GPR68 (as a consequence of degradation of IKAROS1) but also haploinsufficiency of calpastatin, an otherwise inhibitor of the pathway (Fang et al., 2016) (**Figure 1**). Recently, emphasis was placed on the ubiquitination and degradation of CSNK1A1, an inhibitor of TP53, and also part of the ß- catenin destruction-complex (Elyada et al., 2011; Knippschild et al., 2014). Heterozygous loss of this gene, as seen in 5q- MDS leads to an increase in ß-catenin levels and thus, stem cell expansion. Interestingly, complete loss of this protein induces apoptosis as a result of TP53 activation (Schneider et al., 2014) and thus, exposes a vulnerability of 5q- MDS cells to treatment with lenalidomide. However, more recent findings show that this vulnerability emerges only in MDS cells that previously undergo megakaryocytic differentiation driven by a RUNX1-GATA2 complex. This complex is enabled by the lenalidomide-induced ubiquitination and degradation of Ikaros protein IKZF1(Martinez-Høyer et al., 2020).

Nevertheless, lenalidomide was found to have clinical activity in some patients with LR-MDS without 5q- and even some patients with HR-MDS. While some of these effects can be explained by lenalidomide-dependent improved erythropoietin signaling in MDS cells (Basiorka et al., 2016), broader effects of this drug on the immune and mesenchymal BME are also likely to play a role. To this end, lenalidomide increased CD28 signaling, resulting in augmented T cell costimulation and increased secretion of interferon gamma and IL2 (**Figure 1**) (Stirling, 2001; LeBlanc et al., 2004; Wu et al., 2008). In addition, treatment with lenalidomide results in decreased production of pro-inflammatory cytokines (TNF-a, IL-1, IL-6, IL-12) and increased levels of anti-inflammatory cytokines such as IL10. Altered cytokine milieu may not only be detrimental to the MDS clone but also promote the wellbeing of residual normal hematopoiesis (Kotla et al., 2009; Stahl and Zeidan, 2017). Regarding the impact of lenalidomide on the BME, treatment with lenalidomide decreases levels of CXCL12 production from normal as well as MDS-MSCs (**Figure 1**) (Ferrer et al., 2013). Since levels of CXCL12 are already low in MDS BME, further reduction may be detrimental to the MDS clone but could also explain some of the cytopenias associated with this drug. In addition, lenalidomide like thalidomide is a powerful antiangiogenic agent. In MDS, the bone marrow niche is characterized by increased neoangiogenesis. Lenalidomide reduces marrow vascular density and this histologic effect correlates with decreased disease progression (Stahl and Zeidan, 2017).

While the immunomodulatory and anti-angiogenic properties of lenalidomide are well recognized, their role in the observed clinical benefit for patients with 5q- MDS or non 5q- MDS remains to be further clarified.

### Luspatercept

Anemia and RBC transfusion requirement is a major source of morbidity in patients with MDS. In this aspect, MDS resemble hemoglobinopathies such as β-thalassemia in that ineffective erythropoiesis results not only in refractory anemia but also accumulation of various erythroid precursors and disruption of the BME. TGF-β signaling regulates terminal erythroid maturation and targeting this pathway either by using activin receptor traps (luspatercept, sotatercept), TGF-βR1 tyrosine kinase inhibitor (galunisertib) or targeting SMAD7 *via* miR21 promised improved erythropoiesis in patients with LR-MDS. Initially, FDA approved the use of luspatercept only for the treatment of transfusion-dependent ß-thalassemia. However, in early April 2020, the drug was also approved in transfusion-dependent LR-MDS with ring sideroblasts, after failure of treatment with erythropoiesis stimulating agents. In this category of patients, treatment with luspatercept resulted in more than a third of patients achieving transfusion independence for 8 weeks or longer (Fenaux et al., 2020). These results are consistent with previous clinical studies showing that patients with >15% ring sideroblasts, or with SF3B1 mutations, are most likely to benefit from this therapeutic approach (Platzbecker et al., 2017). In contrast with HMAs and lenalidomide, luspatercept does not impact MDS clonal evolution but rather rescues erythropoiesis in these patients.

MDS cells are characterized by hyperactivity of the TGF-ß signaling, mostly due to SMAD2/3 dependent reduction of SMAD7, a negative-feedback regulator of the pathway (Zhou et al., 2011; Bhagat et al., 2013). A variety of ligands such as activin A, activin B, GDF8, GDF11, and several BMPs can also activate TGF-ß signaling and thus, regulate/dysregulate erythroid maturation (Krolewski et al., 1992).

Luspatercept is a chimera between the extracellular domain of activin receptor and human IgG1 Fc portion serving as a TGFβ ligand trap (**Figure 1**). Though initially thought to exert its activity *via* inactivating GDF11, recent genetic studies called into question this mechanism of action (Guerra et al., 2019). A number of other TGFβ ligands, including BMPs, activins, and GDF8 may play role (Verma et al., 2020). Preventing signaling downstream of these ligands can have a profound impact on the BME. For instance, sotatercept improved hematocrit levels in postmenopausal women, prevented osteoporosis and improved osteolytic lesions in patients with multiple myeloma (Raje and Vallet, 2010). These observations came to underscore the known roles of TGFβ ligands in MSCs osteogenic specification and terminal differentiation. It is currently unknown the impact of luspatercept on the MDS BME. That being said, TGF-ß1 secreted by the MSCs contributes to the pro-inflammatory milieu present in the BME of patients with MDS (**Figure 1**) (Cagliani et al., 2017). Interestingly, levels of TGF-ß ligands are higher in high-risk MDS compared to low-risk disease (Zhao et al., 2012) which may explain the lack of clinical activity of single agent luspatercept in high-risk MDS.

## Emerging Therapies in MDS

### Rigosertib

Rigosertib is a multi-kinase inhibitor with promising activity in a number of malignancies including MDS. Preclinical and early phase clinical trials showed encouraging activity of this drug in patients with MDS that failed hypomethylating agents. Some of these patients achieved partial/complete marrow responses with acceptable hematologic toxicity (cytopenias) and no other significant adverse events (Navada et al., 2018). Thus, this therapeutic approach is now tested in a phase III clinical trial. Initially thought to act as a polo-like kinase 1 (PLK1) inhibitor (Gumireddy et al., 2005), rigosertib is also a powerful phosphatidylinositol 3-kinase (PI3K) (Prasad et al., 2009) and RAS inhibitor (Athuluri-Divakar et al., 2016). Most recently, a CRISPRi-based chemical genetic screen revealed that rigosertib may also act as a microtubule-destabilizing agent binding the same tubulin-site as colchicine (Jost et al., 2017). Rigosertib induces mitotic arrest and subsequent apoptosis in MDS cells (Hyoda et al., 2015). Although *in vitro* studies showed promising activity in AML and MDS cells (Sloand et al., 2007; Olnes et al., 2012; Hyoda et al., 2015), most clinical trials showed no significant improvement in overall survival, and limited hematological improvement (Garcia-Manero et al., 2016). Rigosertib does not impair normal HSPCs functions *in vitro* (Xu et al., 2014) but *in vivo*, it was shown to remodel the bone architecture of young mice due to increased osteoclast numbers. In this model, rigosertib treatment resulted in decreased mass, thickness and numbers of trabecular bone leading to pancytopenia. More so, rigosertib altered the biomechanical properties of MSCs and reduced hematopoiesis-supporting properties of MDS-MSCs (Balaian et al., 2019). In addition, the downregulation of the PI3K/Akt pathway activity, one of the main targets of the drug, is associated with dysfunction of the stromal cells (Falconi et al., 2016) and thus, further impairment of normal hematopoiesis. Since hyper activation of Wnt/ß-catenin signaling in MSCs leads to rapid development of MDS and AML the proposed inhibitory effects of rigosertib may correct the altered BME in patients with MDS. Rigosertib can rescue Akt, ß-catenin, and GSK3a/ß signaling pathways commonly dysregulated in MDS cells (**Figure 1**) (Xu et al., 2014) but it is unclear if these effects hold true *in vivo* in patients with MDS treated with this drug (Stoddart et al., 2017). Even less is known about the biological effects of rigosertib on these pathways in MDS-MSCs. Most intriguing, the newly described role of rigosertib in microtubule assembly may have significant implications for hematopoiesis–MSCs interactions given the recent reports that mitochondria can be transferred between MSCs and malignant cells *via* tunneling nanotubules. Inhibition of microtubule assembly, as seen during treatment with vincristine, and potentially rigosertib can destabilize tunneling nanotubules with profound impact on the survival and metabolic profile of malignant cells (Moschoi et al., 2016; Forte et al., 2019). To what extent this mechanism plays a role in the biological activity of rigosertib remains to be evaluated.

## Is There a Role for ATRA in MDS?

Multi lineage cytopenias is the hallmark of MDS and yet bone marrow cellularity is typically increased in these patients. Abnormal myeloid and erythroid elements in various stages of differentiation dominate the histology of patients with MDS. Lack of final maturation of these cells is the root for most mortality and morbidity in MDS. Therapeutic interventions to promote final maturation of the dysplastic cells have been met with some success in the case of erythroid maturation and improvement in RBC transfusion needs in response to erythropoietin stimulating agents (ESA) [for a review on the topic see (Park et al., 2019)].

Vitamin A plays an essential role in hematopoiesis. The impairment of vitamin A pathway, due to abnormal retinoic acid receptor alpha signaling, results in a block in myeloid differentiation and development of APL (Evans, 2005). In this case, treatment with high levels of ATRA, the active compound of vitamin A, overcomes this differentiation block and promotes final maturation of the malignant cells with emergence of neutrophils. Thus, a similar approach was attempted in MDS. To this end, ATRA induces G0/G1 cell cycle arrest of MDS cell lines *in vitro via* downregulation of CDK4, CDK6, cyclinD3, and cyclinD1 (Huang et al., 2004). Similarly, 4-Amino-2-Trifluoromethyl-Phenyl Retinate (ATPR), a synthetic retinoid, inhibits proliferation and promotes apoptosis of MDS cells *in vitro*, likely *via* upregulation of TP53 (Du et al., 2018). While single agent ATRA had only minimal effect in patients with MDS, co-administration with recombinant erythropoietin was effective in 40% of patients with LR-MDS and low erythropoietin levels (Itzykson et al., 2009). Treatment with ATRA has been tried in combination with valproic acid, vitamin E, vitamin D, interferon-α, and other therapeutic interventions but unfortunately showed discouraging results in MDS (Hofmann et al., 1999; Cortes, 2005; Giagounidis et al., 2005; Zhang et al., 2006). Most recently, impaired local pharmacokinetics of ATRA in the BME has been proposed to account for the observed discrepancy between *in vitro* sensitivity of MDS cells to ATRA and relative lack of clinical efficacy (Hernandez et al., 2020). To this end, ATRA is oxidized and inactivated by CYP26, a member of cytochrome P450 enzymes. Though initially known to control systemic ATRA levels *via* their hepatic function, CYP26 enzymes were recently shown to be expressed by BM MSCs (Ghiaur et al., 2013; Su et al., 2015; Alonso et al., 2016; Hernandez et al., 2020). More so, stromal CYP26 is essential to maintain normal and malignant stem cell activity (Ghiaur et al., 2013; Su et al., 2015; Alonso et al., 2017). Interestingly, treatment with ATRA directly upregulates stromal CYP26 (Hernandez et al., 2020) and thus, results in hyper-protective niches in the BME (**Figure 1**). Inhibition of stromal CYP26 was able to sensitize malignant cells to retinoid induced differentiation in the presence of BME (Su et al., 2015). Similarly two synthetic CYP26 resistant retinoids (IRX195183 and Tamibarotene) can bypass stromal protection and induce myeloid differentiation in some AML and multiple myeloma cells (Alonso et al., 2016; Hernandez et al., 2020). These novel retinoids have shown biological activity in preliminary results from two ongoing clinical trials that also enrolled patients with MDS. The full clinical impact of these tools remains to be seen, particularly in combination therapies with ESA or other MDS targeting agents.

## Conclusion

MDS is a complex disease with great heterogeneity and poorly understood pathogenesis. Although progression to AML is a feared complication, most of morbidity and mortality stems from multi lineage cytopenias and subsequent infections, bleeding complications, and long term toxicities from frequent transfusions. It is only beginning to be explored how the malignant stem cells out compete their healthy counterparts. The role of bone marrow microenvironment in this process is becoming front and center. By now, it is clear that MDS-MSCs are profoundly altered and contribute to maintaining the dysplastic clone while suppressing residual normal hematopoiesis. The exact mechanisms by which abnormal MDS-MSCs contribute to disease homeostasis are only now being explored.

This is an exciting time in our understanding of MDS. Innovative preclinical models coupled with wide use of next generation sequencing in patients with MDS led to rapid development of new therapeutic tools. A multitude of drugs are in various stages of clinical development. We are now testing APR246 for TP53 mutant disease, IDH inhibitors for IDH mutant disease, as well as CDK9 and Bcl2 inhibitors. These drugs have already shown activity in subtypes of AML and are bound to change our understanding of MDS. Other approaches, such as immune checkpoint inhibitors, Hypoxia-inducible factor prolyl hydroxylase inhibitor, Hedgehog inhibitors, and splicing modulators are testing new biological concepts. Nevertheless, while we are zooming in on the biological effects of these drugs on the malignant clone, one should not loose site that MDS is a disorder in which the entire bone marrow (hematopoietic and non-hematopoietic) is profoundly perturb. Thus, a system biology approach to not only the pathophysiology of the disease but also to understanding the response or lack thereof to these new agents holds promise to better the clinical outcomes for patients with MDS.

## Author Contributions

PT, SP, CT, GG—designed the project. PT, GG—wrote the first draft of the manuscript. DD—contributed to critical discussion.

## Funding

The project was funded by K08 HL127269 (GG), R03 HL145226 (GG), P01CA225618 (GG), and P30 CA006973.

## Conflict of Interest

The authors declare that the research was conducted in the absence of any commercial or financial relationships that could be construed as a potential conflict of interest.
